# PTEN expression is consistent in colorectal cancer primaries and metastases and associates with patient survival

**DOI:** 10.1002/cam4.97

**Published:** 2013-06-10

**Authors:** Chloe E Atreya, Zaina Sangale, Nafei Xu, Mary R Matli, Eliso Tikishvili, William Welbourn, Steven Stone, Kevan M Shokat, Robert S Warren

**Affiliations:** 1 Department of Internal MedicineDivision of Hematology/OncologyHelen Diller Family Comprehensive Cancer Center, University of CaliforniaSan Francisco, California, USA; 2 Myriad Genetics Inc.Salt Lake City, Utah, USA; 3 Department of SurgeryDivision of Surgical OncologyHelen Diller Family Comprehensive Cancer Center, University of CaliforniaSan Francisco, California, USA; 4 Howard Hughes Medical InstituteSan Francisco, California, USA; 5 Department of Cellular and Molecular Pharmacology, University of CaliforniaSan Francisco, California, USA; 6 Department of Chemistry, University of CaliforniaBerkeley, California, USA

**Keywords:** Biomarker, colorectal cancer, concordance, PTEN, survival

## Abstract

Phosphatase and tensin homologue deleted on chromosome 10 (PTEN) negatively regulates the phosphoinositide-3-kinase (PI3K) signaling pathway. In colorectal cancer (CRC), observed frequencies of loss of PTEN expression, concordant expression in primary tumors and metastases, and the association of PTEN status with outcome vary markedly by detection method. We determined the degree to which PTEN expression is consistent in 70 matched human CRC primaries and liver metastases using a validated immunohistochemistry assay. We found loss of PTEN expression in 12.3% of assessable CRC primaries and 10.3% of assessable liver metastases. PTEN expression (positive or negative) was concordant in 98% of matched colorectal primaries and liver metastases. Next we related PTEN status to mutations in RAS and PI3K pathway genes (*KRAS*, *NRAS*, *BRAF,* and *PIK3CA*) and to overall survival (OS). PTEN expression was not significantly associated with the presence or absence of mutations in RAS or PI3K pathway genes. The median OS of patients whose tumors did not express PTEN was 9 months, compared to 49 months for patients whose tumors did express PTEN (HR = 6.25, 95% confidence intervals (CI) (1.98, 15.42), *P* = 0.0017). The association of absent PTEN expression with increased risk of death remained significant in multivariate analysis (HR = 6.31, 95% CI (2.03, 17.93), *P* = 0.0023). In summary, PTEN expression was consistent in matched CRC primaries and in liver metastases. Therefore, future investigations of PTEN in metastatic CRC can use primary tumor tissue. In patients with liver-only metastases, loss of PTEN expression predicted poor OS.

We observed concordant PTEN expression in 98% of colorectal cancer (CRC) primary and liver metastasis pairs using a validated immunohistochemistry assay. Consistent PTEN expression at both disease sites is significant because tumor tissue is usually available from CRC primaries but not metastases. Loss of PTEN expression associated with poor survival of CRC patients with liver-only metastases.

## Introduction

Two signaling pathways downstream of the epidermal growth factor receptor (EGFR) are dysregulated in the majority of colorectal cancers (CRC): the mitogen-activated protein kinase (MAPK) and the phosphoinositide-3-kinase (PI3K) pathway. Activating mutations in *KRAS* and *BRAF* (MAPK pathway) and *PIK3CA* affect prognosis and/or response to EGFR-directed monoclonal antibodies and other molecularly targeted agents 1–4. Studies investigating the significance of the phosphatase and tensin homologue deleted on chromosome 10 (PTEN) tumor suppressor gene, a negative regulator of the PI3K pathway 5–6, in CRC have yielded inconsistent results 2–13.

Two previous studies reported negative correlations between loss of PTEN in CRC metastases and response to cetuximab, progression-free survival, and/or overall survival (OS). Comparable correlations were not observed following analysis of matched primary tumors 7–8. The concordance of PTEN expression in paired CRC metastases and primaries ranged from 49% to 89% in six studies, the median being 68% 7–13. By contrast, with sensitive detection methods, mutations in *KRAS*,* BRAF,* and *PIK3CA* are about 95% concordant in paired CRC metastases and primary tumors 13–17. The lack of consistent PTEN expression in CRC primaries and metastases (low PTEN concordance) could result from late or heterogeneous loss of PTEN expression, but could also be attributable to the lack of a reproducible immunohistochemistry (IHC) assay 18–19. High PTEN concordance with a validated assay would establish PTEN as a molecular marker meriting further investigation in large numbers of available primary tumors from patients with metastatic CRC. Prospective evaluation of PTEN expression in primary tumors may also guide selection of patients for clinical trials with PI3K-p110β inhibitors 20,21.

In sporadic CRC, loss of PTEN protein expression occurs via several mechanisms, most commonly epigenetic silencing by promoter methylation, and less often mutation or allelic loss at the 10q23 locus 10–25. Although PTEN mutations are easily detectable, they are enriched in hypermutated tumors and may or may not cause loss of protein expression 12–24. IHC is the most effective way to assay for loss of PTEN expression by any mechanism and to distinguish expression in tumor cells from that in adjacent normal cells 26–27. PTEN poses several challenges for IHC analysis, however. Because PTEN is ubiquitous, there are no true negative internal tissue controls. Furthermore, PTEN antibodies differ in specificity, and neither protocols for antibody use nor interpretation of staining results are standardized 27. In nine CRC studies, the frequency of absent PTEN expression ranged from 20% to 49%, the median being 40% 4–32.

An optimized PTEN IHC assay was recently developed through rigorous testing of antibody specificity and selectivity using samples with known molecular alterations in PTEN, paired with a reproducible method of interpretation, and validated 27. Our first objective was to determine the degree of concordance of PTEN expression in matched pairs of CRC primaries and liver metastases using this reliable assay. Next, we associated PTEN expression with mutations in the MAPK and PI3K pathways and with the survival of patients treated for liver-only CRC metastases.

## MATERIALS AND METHODS

### Patients

In accord with a research protocol approved by the University of California San Francisco (UCSF) Committee on Human Research, all patients who underwent resection of a CRC and resection or biopsy of a corresponding liver metastasis at UCSF between 1990 and 2010 were eligible for inclusion in this study. Patients with unresectable liver metastases underwent liver tumor biopsy at the time of implantation of a pump for hepatic arterial infusion of floxuridine 33. UCSF pathology reports were used to select tumor-containing formalin-fixed paraffin-embedded (FFPE) tissue blocks. All patients with sufficient amounts of matched tumor tissue were included. Clinicopathologic characteristics, treatments received, and outcomes data were stored in a secure UCSF Surgical Oncology database. No patient-identifying information was shared between UCSF and Myriad Genetics. All unused tissue and extracted DNA were returned to UCSF.

### Immunohistochemistry

PTEN IHC was performed at Myriad Genetics, Inc. (Salt Lake City, UT) using rabbit monoclonal antibody 138G6 (Cat. 9559, Cell Signaling Technology, Danvers, MA), and a protocol optimized by Sangale et al. 27. Briefly, 5 μm sections cut from FFPE tumor blocks were mounted on charged glass slides and PTEN IHC was completed on the Discovery XT platform (Ventana Medical Systems, Tucson, AZ). The assay was carried out using a 1:100 primary antibody dilution, incubated for 36 min without heat and visualized with the ChromoMap DAB detection kit with OmniMap (Ventana Medical Systems) anti-rabbit HRP. Slides were then counterstained and cover slipped. PTEN expression was interpreted by a pathologist (Z. S.) blinded to whether colon and liver tumors originated from the same patient. A binary scoring system was used: negative PTEN expression was defined as no staining in greater than or equal to 90% of tumor cells, whereas positive PTEN expression was defined as staining in more than 10% of tumor cells. Because PTEN is expressed in both the nucleus and cytoplasm, a negative sample must exhibit no staining in either compartment in at least 90% of the tumor cells. For positive samples, only cytoplasmic expression was scored. The entire tissue section was evaluated. Internal controls (stromal elements) were used to assess tissue integrity and external negative controls ensured assay quality. A comparison of our assay to others in the literature is in Supporting Information.

### Microscopic imaging

Slides were scanned into an Aperio ScanScope CS (Vista, CA) image capture device at a 20× equivalent objective. ImageScope software annotated and captured the digital slide images. Details are in Supporting Information.

### Genomic DNA isolation

One 5 μm hematoxylin and eosin (H&E) slide and three to five subsequent 10 μm unstained slides were made from each FFPE tumor block. A pathologist marked the tumor on the H&E slide to guide macrodissection of unstained slides. A razor blade was used to macrodissect the tumor; paraffin was removed using xylene; and the sections were rehydrated in a graded series of ethanol baths. Tumor DNA was extracted using a QIAmp DNA FFPE tissue kit (#56,404, Qiagen, Germantown, MD) per the manufacturer's protocol with the following modifications: extension of the 56°C proteinase K incubation to 72 h with addition of new proteinase every 24 h. After isolation, tumor DNA was diluted to 3 ng/μL in TE buffer (10 mmol/L Tris-HCl, 0.1 mmol/L ethylenediaminetetraacetic acid, pH 8.0).

### Mutational analysis

Peptide nucleic acid (PNA) clamps were used to selectively amplify genomic DNA containing hotspot mutations in *KRAS* (G12 and G13) 34, *PIK3CA* (E542, E545, and H1047), and *BRAF* (V600). PCR products were sequenced in the forward and reverse directions using Sanger Big Dye Primer Sequencing chemistry on a MegaBACE 4500 (GE Healthcare, Buckinghamshire, U.K.) at Myriad Genetics, Inc. Each mutation was confirmed with two independent sequencing reactions. Myriad investigators were blinded to which colon and liver tumors originated from the same patient.

Following review of the Myriad results at UCSF, 52 paired samples, including cases with a primary/metastasis mutational mismatch and all samples that were wild-type for *KRAS* and *BRAF*, were further analyzed in the UCSF Genome Analysis Core using quantitative mass spectrometry-based multiplex technology (Sequenom, San Diego, CA). Standard Sequenom platform protocols and a colorectal cancer-specific mutational panel (ColoCarta) were employed 35. iPLEX well-6, containing *KRAS*-Q61L and *HRAS*-Q61L primers, was omitted. Data analysis was accomplished via MassArray Typer Analyzer software (Sequenom) and manual review.

### Statistical analysis

Univariate Cox proportional hazards (Cox PH) regression models and a multivariate Cox PH model estimated relative risks and tested associations of biomarkers, clinical features, and demographic characteristics with OS. OS was defined as the time from diagnosis of primary CRC to death from any cause. Observations were censored on the date of last follow-up. Variables associated with OS in the univariate analysis at the 5% significance level were considered as potential predictors in the multivariate analysis. We used a forward stepwise regression approach to construct the multivariate model, in which a new variable was added only if it yielded a *P*-value less than 0.05. Hazard ratios (HR) and their relative profile likelihood 95% CIs were used to estimate relative risks. All *P*-values for testing associations were based upon partial likelihood ratio chi-square statistics. Survival curves were estimated using the product-limit (Kaplan–Meier) method. A test for linear trend in OS, for increasing values of a given ordinal categorical variable, was carried out with a univariate Cox PH model, using equally-spaced scoring in the categorical variable.

Fisher's exact test assessed the association between categorical variables where, whenever applicable, the appropriate strength of the association was expressed by an odds ratio (OR) and relative 95% CIs. The exact binomial test method was adapted to construct the appropriate 95% CI for a single population proportion. A comparison of the concordance between primary CRC and liver metastases for two binary categorical variables was carried out by way of the exact McNemar's test of the null hypothesis of homogeneity between the margins of the appropriate two-way contingency table; this comparison of concordance upon two three-level categorical variables was carried out by way of one million Monte Carlo resamples within the Stuart-Maxwell generalized McNemar marginal homogeneity test 36–37.

Statistical significance was set at the 5% level, where two-sided alternative hypotheses were considered for all tests of significance. Analyses were conducted within the R software, version 2.15.1 (R Foundation for Statistical Computing, Vienna, Austria) at Myriad Genetics, Inc.

## Results

### Clinical characteristics

We collected 145 FFPE CRC tumor blocks from 70 patients. Seven patients were excluded due to insufficient primary tumor tissue. Clinical and demographic characteristics of the 63 remaining study patients are summarized in Table 1 and detailed in Table S1. The majority of patients had stage IV disease at presentation, and all had liver-only metastases. In two-thirds of cases, the paired specimens were synchronous. Of metachronous pairs, colectomy preceded metastectomy in all but two cases. No patient received EGFR-targeted monoclonal antibodies prior to surgery, but four patients were subsequently treated with cetuximab.

**Table:** Clinical and demographic characteristics for the cohort of 63 patients

Characteristic	Summary measure
Age (years) at diagnosis: mean ± SD	57.2 ± 11.7
Gender (males): *N* (%)	33 (52.4)
Stage at diagnosis: *N* (%)	
I	0 (0.0)
II	5 (7.9)
III	5 (7.9)
IV	53 (84.2)
Tumor location: *N* (%)	
Right colon	24 (38.1)
Left colon	15 (23.8)
Rectum	24 (38.1)
Tumor grade (high^1^): *N* (%)	18 (28.6)
Mucinous features (present): *N* (%)	14 (22.2)
Surgical timing (synchronous^2^): *N* (%)	41 (65.1)
Chemotherapy^3^ before surgery: *N* (%)	
No	41 (65.1)
Yes, before liver surgery only	7 (11.1)
Yes, both colon and liver surgeries	15 (23.8)
Liver metastases: *N* (%)	
Resectable	35 (55.6)
Unresectable	28 (44.4)

1 Includes intermediate/high grade.

2 Colon and liver tumor samples collected within 3 months of each other. Twenty-seven patients underwent a single operation to remove both the primary and metastasis (true synchronous).

3 5-Fluorouracil-based chemotherapy, details in Table S1.

Of the 63 patients comprising our cohort, 48 (76%) died over the course of the study. The median follow-up time for the entire cohort was 28 months (Table S1). The median OS of patients with unresectable liver metastases was 18 months, compared to 58 months when liver metastases were resected with curative intent (HR = 7.68, 95% CI (3.81, 14.61), *P* = 9.0 × 10^−10^). The median OS for the entire study cohort was 33 months (Table S1 and Fig. S1). Biopsies of unresectable liver metastases were obtained during a palliative procedure now infrequently used at UCSF.

### Concordance of PTEN expression in paired CRC primaries and liver metastases

**Figure 1:**
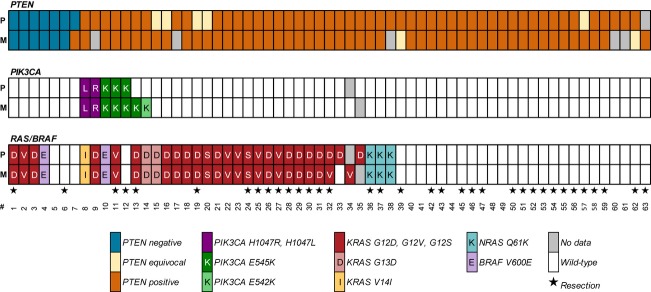
Concordance of PTEN expression, *PIK3CA*, and *KRAS/NRAS/BRAF* mutations in 63 pairs of primary and metastatic colorectal carcinomas. The top rows represent the primary tumors (P) and the bottom rows represent the liver metastases (M). Patient number is listed below. Stars designate patients who underwent liver resection with curative intent. *PIK3CA* E545 and E542 mismatches in primaries and metastases from patients 13 and 14, respectively, were confirmed by Sequenom (Fig. S2). *KRAS* V14I is a rare but transforming mutation detected in the tumors of patient 8 using PNA clamps designed to amplify *KRAS* codon 13 mutations. The *KRAS* G12D mutation in patient 33's colon primary was only detectable using PNA clamping (not Sequenom). Apart from *NRAS* mutations in three *KRAS/BRAF* wild-type tumors, no additional or different mutations were identified with Sequenom versus PNA clamping.

**Figure 2:**
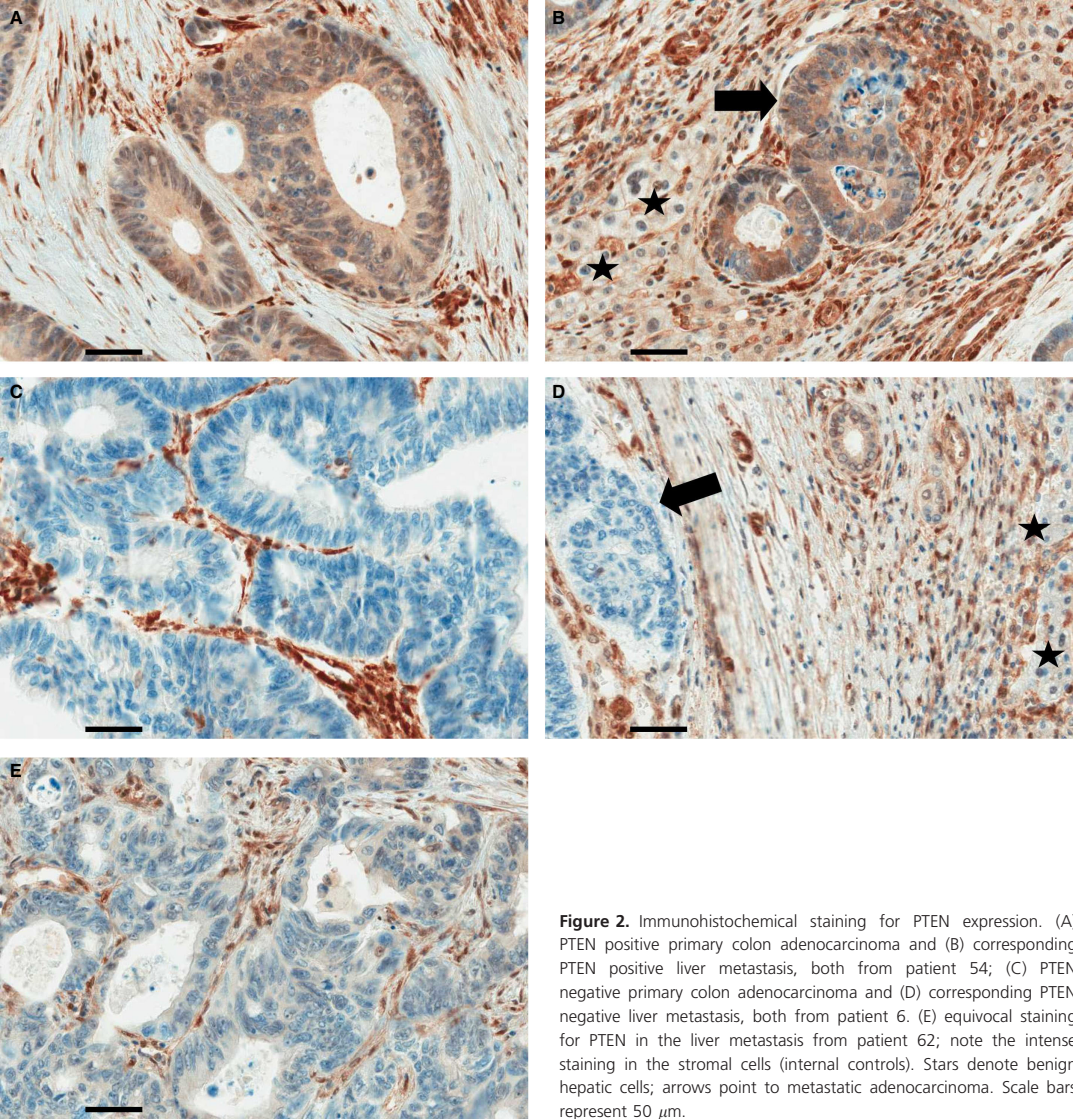
Immunohistochemical staining for PTEN expression. (A) PTEN positive primary colon adenocarcinoma and (B) corresponding PTEN positive liver metastasis, both from patient 54; (C) PTEN negative primary colon adenocarcinoma and (D) corresponding PTEN negative liver metastasis, both from patient 6. (E) equivocal staining for PTEN in the liver metastasis from patient 62; note the intense staining in the stromal cells (internal controls). Stars denote benign hepatic cells; arrows point to metastatic adenocarcinoma. Scale bars represent 50 μm.

Loss of PTEN expression was observed in 7 of 62 assessable CRC primaries (12.3%) and 6 of 58 assessable liver metastases (10.3%) (Figs. 1 and 2). Uninterpretable results due to poor internal control staining were more common in liver metastases than in primary CRC. This was sometimes secondary to poor tissue morphology, including diffuse necrosis, and was not associated with exposure to chemotherapy (Table S1; OR = 2.87, 95% CI (0.29, 144.3), *P* = 0.656). Uninterpretable results were distinguished from equivocal staining, which was defined as diffuse, very weak staining of tumor cells that could represent either a weak positive or a nonspecific negative result (Fig. 2E). In two of four cases where multiple, synchronous liver lesions from the same patient were available, staining was equivocal in some metastases and positive in others (reported as positive, Fig. 1). The percent of cells staining positive for PTEN followed a bimodal distribution, with the majority of tumors exhibiting PTEN expression in 0% or 100% of cells (Table S1B).

After exclusion of cases with uninterpretable or equivocal PTEN staining, 50 CRC primary/liver metastasis pairs remained (Fig. 1). PTEN expression (positive or negative) differed in only one pair, corresponding to 98% concordance (95% CI for true proportion of concordance, [89.3%, 99.9%]) (Table 2). Patient 7, with discordant PTEN staining, had surgery to remove the CRC primary and biopsy of liver metastases within 3 months, without intervening chemotherapy. Two metastases were sampled: staining was positive in one lesion and equivocal in the other, with mostly necrotic tissue and few tumor cells (not shown).

**Table:** Concordance of PTEN, PIK3CA, RAS, and BRAF in paired CRC primaries and liver metastases.^1^

		CRC primaries										
PTEN						PIK3CA									RAS/BRAF										
Neg	Pos	Equ		Wt	Mut		Wt	Mut
Liver metastases	PTEN Neg	6	0	0	PIK3CA Wt	54	0	RAS/BRAF Wt	29	1
	PTEN Pos	1	43	5	PIK3CA Mut	2	5	RAS/BRAF Mut	0	31
	PTEN Equ	0	2	0						
Concordance		98.0% (86.0%)^2^						96.7%									98.4%										

1 Neg, negative; Pos, positive; Equ, equivocal; Wt, wild-type; Mut, mutant.

2 Parenthetic value is concordance in PTEN expression when equivocal expression was considered an observable category of PTEN status.

If only patients with uninterpretable PTEN staining were excluded, 57 CRC primary/liver metastasis pairs remained (Fig. 1). PTEN expression (positive, negative or equivocal) differed in eight pairs (Fig. 1), corresponding to 86.0% (49/57) concordance (95% CI for proportion of concordance, [74.2%, 93.7%]). This concordance is the most conservative estimate for these data, as it assumes that every equivocal PTEN measurement disagrees with the PTEN status of the matched sample.

### PTEN expression is not associated with mutation of *PIK3CA* or *KRAS/NRAS/BRAF*

Sanger sequencing with PNA clamping of wild-type alleles and the Sequenom platform were used to assay for mutations in *PIK3CA*,* KRAS*,* NRAS,* and *BRAF* (Fig. 1). The frequency of *PIK3CA* mutations was 8% in colorectal primaries (5/62) and 11% (7/62) in liver metastases, with 96.7% concordance (59/61 matched pairs; 95% CI for proportion of concordance, [88.7%, 99.6%]) (Table 2). In two cases, a subpopulation of tumor cells with a mutation in exon 9 of *PIK3CA* was detected only in the metastasis (Fig. S2). The frequency of MAPK pathway mutations in *KRAS*,* NRAS* or *BRAF*, was 53.2% (33/62) in primaries and 51.6% (32/62) in metastases. MAPK pathway mutations were 98.4% concordant in matched primaries and metastases (60/61; 95% CI for proportion of concordance, [91.2%, 100.0%]) (Table 2).

Overall, 60.3% of tumors exhibited at least one aberration in the PI3K or the MAPK pathway and 16% of tumors contained aberrations in both pathways (38/63 and 10/63 patients, respectively; Fig. 1). Similarly, 57% of samples in which PTEN was not expressed (4/7) exhibited a mutation in either *KRAS* or *BRAF*. Concomitant absence of PTEN staining and the presence of mutant *PIK3CA* were never observed in the same tumor, but this result was not statistically significant. In fact, there was no statistically significant association between PTEN expression and mutation of *PIK3CA* or *KRAS*/*NRAS*/*BRAF* in CRC primaries or liver metastases (Tables S2 and S3).

### Association of PTEN expression with OS

**Figure 3:**
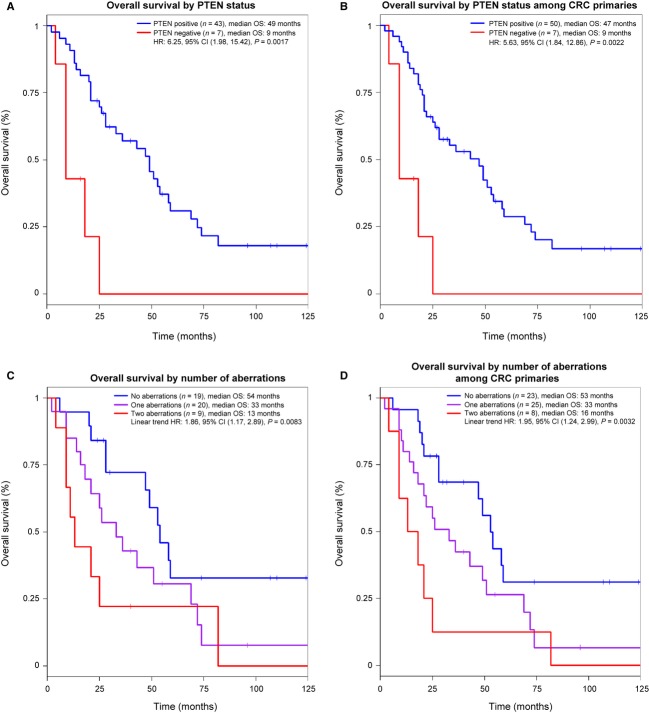
Kaplan–Meier estimates of overall survival related to: (A) PTEN expression in matched colorectal primaries and liver metastases; (B) PTEN expression in colorectal primaries; (C), number of aberrations in PTEN, *PIK3CA* and/or *KRAS/NRAS/BRAF* in matched colorectal primaries, and liver metastases; and (D) number of biomarker aberrations in colorectal primaries.

The median OS of patients with tumors that did not express PTEN was 9 months from diagnosis (Table 3), compared to 49 months for patients with PTEN positive matched primaries and metastases (HR = 6.25, 95% CI [1.98, 15.42], *P* = 0.0017) (Fig. 3A and Table S1). Results were similar for OS by PTEN status among all assessable CRC primaries (HR = 5.63, 95% CI [1.84, 12.86], *P* = 0.0022) (Fig. 3B). Of the seven patients whose tumors exhibited loss of PTEN expression, 5 (71.4%) had unresectable liver metastases (95% CI for proportion of unresectable liver metastases [29%, 96.3%], *P* = 0.453) (Table 3). Patient 6, who underwent liver resection and has survived over a year, carries a diagnosis of hereditary nonpolyposis CRC. Patient 7, with PTEN staining in the metastasis, but not the primary, survived 9 months (Table S1).

**Table:** Association of molecular markers with resectable liver metastases and survival

Molecular markers	Number of patients^1^	Liver resections^2^	Median OS (months)
	63 (100.0%)	35 (55.6%)	33
Number aberrant^3^			
None	19 (30.2%)	14 (73.7%)	54
One	20 (31.7%)	13 (65.0%)	33
Two	9 (14.3%)	3 (33.3%)	13
Unknown	15 (23.8%)	5 (33.3%)	23
PTEN negative	7 (11.1%)	2 (28.6%)	9

1 Stated figures are *N* (percent of all patients).

2 Stated figures are *N* (percent of patients within a given row). Patients with unresectable metastases underwent liver biopsy.

3 *P*-value = 0.052, for test of linear trend in proportion of liver resections for increasing number of aberrations; cP-value = 0.0083, for test of linear trend in OS for increasing number of aberrations.

Unknown = PTEN was positive in some tissue type and PTEN was either uninterpretable or equivocal in the paired tissue; or, PIK3CA/RAS/BRAF was wild-type in some tissue and the appropriate genetic data was missing in the paired tissue.

Forty-eight patients had complete biomarker data (PTEN expression; *PIK3CA*,* KRAS*,* NRAS*, and *BRAF* mutation status) from their CRC primary and liver metastasis (Fig. 1). In these patients, there was a decreasing linear trend in the odds of liver metastases being resected as the number of biomarker aberrations increased (OR = 0.45; 95% CI [0.18, 1.01], *P* = 0.052) (Table 3). There was an increasing trend in the risk of death as the number of biomarker aberrations increased (HR = 1.86, 95% CI [1.17, 2.89], *P* = 0.0083) (Table 3 and Fig. 3C).

When the 56 patients with complete biomarker data from their CRC primary were considered, results were similar to those described above. In these 56 patients, there was again a decreasing linear trend in the odds of liver metastases being resected as the number of biomarker aberrations increased (OR = 0.42; 95% CI [0.18, 0.93], *P* = 0.033). There was an increasing trend in the risk of death as the number of biomarker aberrations increased (HR = 1.96, 95% CI [1.24, 2.99], *P* = 0.0032) (Fig. 3D).

In the subcohort of patients with unresectable liver metastases, PTEN expression remained associated with OS (HR = 3.81, 95% CI [1.15, 11.21], *P* = 0.030) (Fig. S3A). Identification of any aberration in the PI3K or MAPK pathway was not significantly related to survival of patients with unresectable or resected liver metastases (Fig. S3B and C).

### Multivariate analysis

Variables significantly associated with OS in a univariate Cox PH regression model (Table S4) were considered in multivariate model construction (Table S5). Although the number of aberrations was a significant predictor of OS in the univariate analysis, we excluded it from the multivariate analysis because of its strong association with PTEN expression (*P* = 0.007). After adjusting for age, liver resection status, and the presence of a *PIK3CA* or *RAS*/*BRAF* mutation, PTEN loss remained significantly associated with an increased risk of death (HR = 6.31, 95% CI [2.03, 17.93], *P* = 0.0023) (Table S5).

## Discussion

Selecting patients who may benefit from a specific targeted therapy requires identification of biomarkers in tumor samples. A practical challenge is intertumor, or even intratumor, heterogeneity that has been shown to exist in several cancer types 18–38. Ideally, the aberration which directs choice of targeted therapy would be concordant across multiple tumor sites in the same patient for two reasons: (1) sampling at one tumor site would likely be representative of tumors at other sites, and (2) consistency across tumor sites suggests that an aberration might be retained because it is important for some aspect of tumor biology.

Here, using a validated IHC assay 27, we show that PTEN expression (positive or negative) is 98% concordant in matched CRC primaries and liver metastases. The absence of PTEN expression associated with diminished OS. Similar results were obtained in the subset of patients with complete molecular marker data in paired tissues, and in CRC primaries. This observation indicates that future investigations of PTEN in metastatic CRC can utilize primary tissue and will facilitate incorporating PTEN expression status into treatment decisions.

Assessment for loss of a tumor suppressor protein is more challenging than detection of an activating mutation. Our findings contrast with previous reports of the frequency of PTEN loss and concordance in CRC primaries and metastases, averaging at 36% 4–32 and 67% 7–13, respectively. Discrepancies relate to use of different PTEN antibodies and consideration of staining intensity in alternative scoring systems. The staining cut-off for defining PTEN-positive tumors did not play a major role because most samples exhibited PTEN expression in 0% or 100% of tumor cells. Our antibody and scoring system were chosen based on specificity and reproducibility 27. If, as observed in this study, roughly 10% of tumors in prior analyses were “true negatives,” this would account for the higher estimates of discordance. Our observed level of concordance of PTEN expression parallels that found with activating mutations in PI3K and MAPK pathway genes 13–17.

A principal limitation of this study, common to investigations requiring matched CRC primaries and metastases, is the small sample size. After exclusion of primary tumors with insufficient tissue and uninterpretable or equivocal PTEN staining, 80% of patients (56/70) were included in the final analyses. Additionally, this is a retrospective study involving a heterogeneous group of patients, in particular patients with both unresectable and resectable liver metastases. All CRC patients included in this study represent a particularly fit subset, with liver-only metastases, preserved liver function, and a good performance status defining their surgical candidacy. Number, size, and location of liver metastases determine resectability. The median OS was 1.5 years for patients with unresectable liver metastases treated with hepatic arterial infusion and almost 5 years for patients who underwent resection with curative intent, similar to what has been reported elsewhere 33–39. The median OS among patients with tumors that did not express PTEN was only 9 months.

Although we found no statistically significant association between PTEN expression and PI3K or MAPK pathway gene mutations, this may be due to the limited sample size. Of the seven patients with tumors that did not express PTEN, none exhibited mutation of *PIK3CA*, whereas four harbored a *KRAS* or *BRAF* mutation. Some, but not all, reports have concluded that *PIK3CA* mutations and PTEN loss are mutually exclusive in breast and other cancers 26–42. Conversely, *RAS*/*BRAF* mutations may be slightly more common in the setting of loss of PTEN expression, as has been documented for *KRAS* and *PIK3CA* mutations in CRC 43–44. The number of aberrations in PTEN, *PIK3CA*,* KRAS*,* NRAS,* and *BRAF* was inversely correlated with OS.

As with PTEN loss, *BRAF* mutation predicts unfavorable outcome and is enriched in the setting of microsatellite instability (MSI) 23,24. Whereas, approximately 10% of unselected CRC primaries harbor a *BRAF* mutation, *BRAF* mutations are observed less frequently in liver metastases (3% in this study) 16,17 and MSI is also uncommon. Larger studies using the validated PTEN assay are needed to ascertain the combined prognostic effect of loss of PTEN expression and MSI, as well as the frequency of loss of PTEN expression in unselected CRC primaries compared to the subset of patients with tissue available from a metastatic site.

We expect to gain new insights into the prognostic as well as the predictive significance of PTEN loss from approximately 1000 *KRAS* wild-type CRC primaries collected as part of CALGB/SWOG 80405, a Phase III Intergroup study that assessed cetuximab or bevacizumab in combination with chemotherapy in patients with metastatic CRC. PTEN analysis using the validated IHC assay, along with testing for MSI, *PIK3CA,* and *BRAF* mutations, are among the correlative studies that will be performed on tumor specimens from patients treated in this study. Meanwhile, mounting preclinical evidence suggests that inhibition of PI3K-p110β, a Class IA PI3K isoform that is not itself mutated, may be a promising therapeutic strategy for the treatment of PTEN-deficient tumors 20,21.

## Supporting Information

**Figure S1.** Kaplan–Meier estimates of overall survival of A, all patients in the cohort (*N* = 63) and B, all patients by stratified presence of resectable or unresectable liver metastases.

**Figure S2.** A-B, PIK3CA codon 545 allele frequencies in the primary tumor and liver metastasis from patient 13.

**Figure S3.** Kaplan-Meier estimates of overall survival related to molecular markers in subcohorts of patients with unresectable or resected liver metastases.

**Table S1.** (A) Detailed patient and tumor characteristics. (B) histopathologic characteristics including percent of cells staining positive for PTEN. **Table S2.** Association of PTEN status with mutations in *PIK3CA, RAS*, and *BRAF*, among CRC primaries. **Table S3.** Association of PTEN status with mutations in *PIK3CA, RAS*, and *BRAF*, among liver metastases. **Table S4.** Summary of the fitted univariate Cox PH models. **Table S5.** Summary of the fitted multivariate Cox PH model.
