# Supplementary figures and images for: PTEN expression is consistent in colorectal cancer primaries and metastases and associates with patient survival
Source: Cancer Med. 2013 Jun 10;2(4):496–506. doi: 10.1002/cam4.97 (PMC3799284; doi:10.1002/cam4.97)

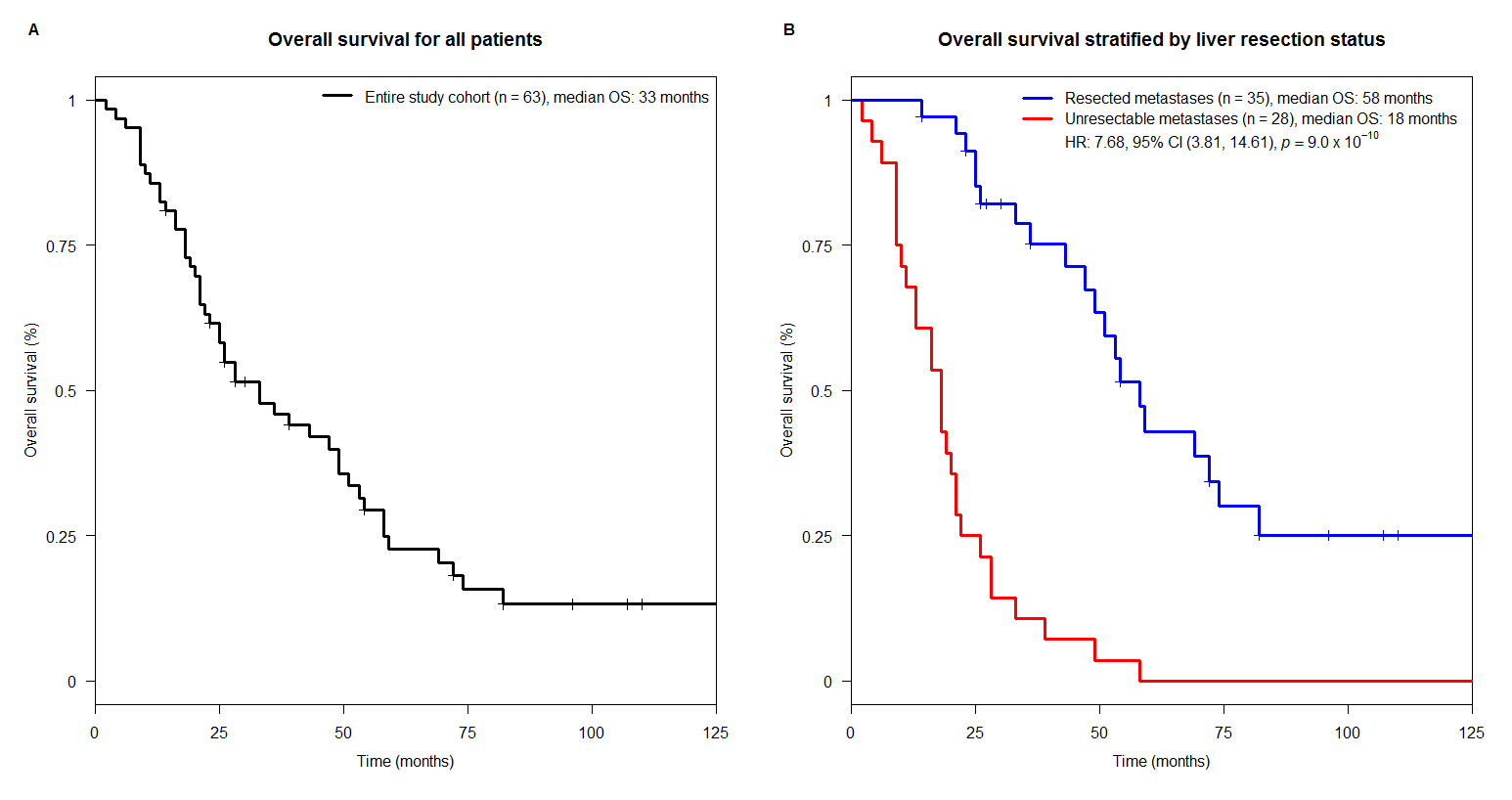

Supplement: Supplementary file 1 — Figure S1. Kaplan–Meier estimates of overall survival of A, all patients in the cohort (N = 63) and B, all patients by stratified presence of resectable or unresectable liver metastases. [file cam40002-0496-SD1.tiff]

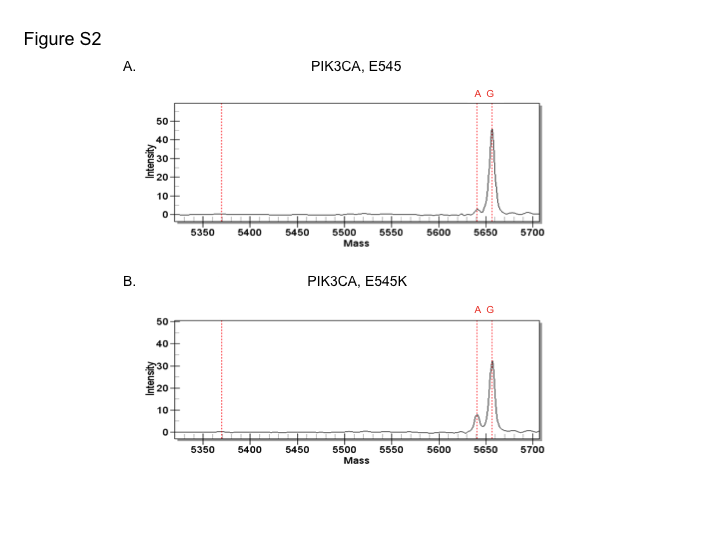

Supplement: Supplementary file 2 — Figure S2. A-B, PIK3CA codon 545 allele frequencies in the primary tumor and liver metastasis from patient 13. [file cam40002-0496-SD2.tiff]

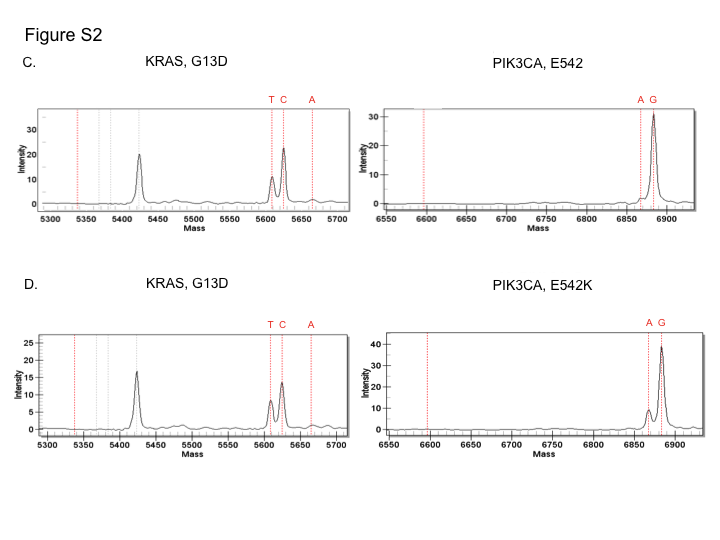

Supplement: Supplementary file 3 — Figure S3. Kaplan-Meier estimates of overall survival related to molecular markers in subcohorts of patients with unresectable or resected liver metastases. [file cam40002-0496-SD3.tiff]

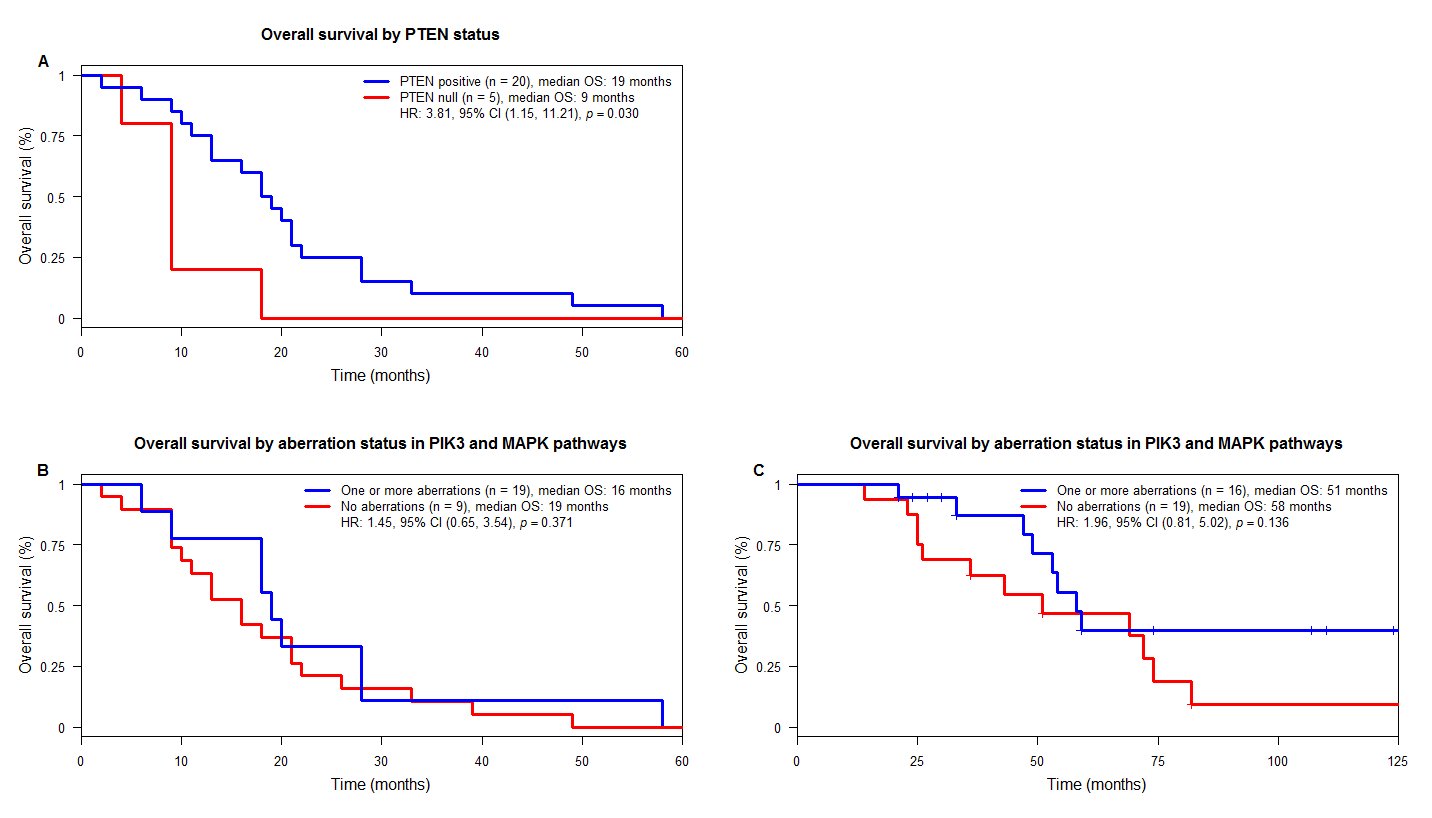

Supplement: Supplementary file 4 [file cam40002-0496-SD4.tiff]
