# Supplementary material for: PTEN expression is consistent in colorectal cancer primaries and metastases and associates with patient survival
Source: Cancer Med. 2013 Jun 10;2(4):496–506. doi: 10.1002/cam4.97 (PMC3799284; doi:10.1002/cam4.97)
Supplement: Supplementary file 5 — Table S1. (A) Detailed patient and tumor characteristics. (B) histopathologic characteristics including percent of cells staining positive for PTEN. Table S2. Association of PTEN status with mutations in PIK3CA, RAS, and BRAF, among CRC primaries. Table S3. Association of PTEN status with mutations in PIK3CA, RAS, and BRAF, among liver metastases. Table S4. Summary of the fitted univariate Cox PH models. Table S5. Summary of the fitted multivariate Cox PH model. [file cam40002-0496-SD5.docx]

**Supplementary Methods**

*PTEN Immunohistochemistry*

Our scoring system is quite different compared to others in the literature.^4, 7, 28^ We use a 10% cut-off for defining positivity, however the majority of tumors exhibit PTEN staining in 0 or 100% of cells (see Table S1B), so changing the cut-off to 50% would not alter the frequency of PTEN null tumors. Perhaps a stronger reason for the variability between studies of PTEN expression is due to differences in antibody specificity.  We evaluated eleven PTEN antibodies and demonstrated that 138G6 (Cell Signaling Technologies) was the most specific of the commercially available antibodies.^27^ We employed a panel of control tissues that were molecularly characterized for PTEN status.  Most of the PTEN antibodies (with the exception of 138G6) were found to have poor sensitivity, specificity, or both.  Staining intensity was considered but was not found to be reproducible. Contributors to the apparent variability in expression (intensity) of PTEN are the reagents, instrumentation vs. manual methods and the inherent biological variation of the normal tissue. We evaluated two scoring systems; a dichotomous method based on a predefined 10% positive cell cut- off and a ‘(percent positive cells) x (intensity 0, 1, 2)’ scoring system. The results of our binary scoring system showed 100% reproducibility between three independent pathologists, while the more complex scoring system was not reproducible.

*Microscopic imaging (Fig. 2)*

1. Magnification: 20x/0.75 Plan Apo Objective
2. Image Compression: JPG2000
3. Image Resolution: 300dpi
4. Color: RGB
5. Image Bit Depth: 8 bits/channel

**Supplementary Tables**

Table S1A: Detailed Patient and Tumor Characteristics

| Patient | Age | Sex | Year | Stage | Location of | Chemotherapy | Synchronous | Liver biopsy | Recurrence | Survival |  |
| --- | --- | --- | --- | --- | --- | --- | --- | --- | --- | --- | --- |
| No. | at dx |  | of dx | at dx | primary^a^ | before surgery | surgeries | or resection | (months) | (months) |  |
| 1 | 64 | F | 1996 | IV | right colon | 5-FU^b^ | yes^c^ | resection | 12 | 25 |  |
| 2 | 62 | M | 2001 | IV | right colon | no | yes | biopsy | n/a | 4 |  |
| 3 | 48 | F | 2002 | IV | right colon | 5-FU, CPT-11 | yes | biopsy | n/a | 9 |  |
| 4 | 49 | F | 1997 | IV | right colon | no | yes | biopsy | n/a | 9 |  |
| 5 | 63 | F | 1999 | IV | right colon | 5-FU, CPT-11 | yes | biopsy | n/a | 18 |  |
| 6 | 46 | M | 2010 | IV | right colon | no | yes^c^ | resection | >3 | >16 |  |
| 7 | 64 | M | 1996 | IV | left colon | no | yes^c^ | biopsy | n/a | 9 |  |
| 8 | 82 | F | 1995 | IV | right colon | 5-FU | yes | biopsy | n/a | 13 |  |
| 9 | 46 | F | 1995 | IV | right colon | no | yes | biopsy | n/a | 18 |  |
| 10 | 47 | M | 2000 | IV | right colon | no | yes | biopsy | n/a | 21 |  |
| 11 | 50 | F | 1995 | IV | right colon | no | yes^c^ | resection | 78 | 82 |  |
| 12 | 76 | M | 2003 | II | rectum | no | no (13 mos) | resection | 9 | 51 |  |
| 13 | 58 | M | 2007 | IV | rectum | no | no (4 mos) | resection | 25 | >40 |  |
| 14 | 63 | M | 1995 | IV | right colon | no | yes | biopsy | n/a | 11 |  |
| 15 | 70 | M | 1991 | IV | left colon | no | yes | biopsy | n/a | 39 |  |
| 16 | 58 | M | 1990 | IV | right colon | 5-FU^b^ | no (18 mos) | biopsy | n/a | 26 |  |
| 17 | 47 | F | 1995 | IV | right colon | no | yes | biopsy | n/a | 22 |  |
| 18 | 73 | M | 1997 | IV | right colon | no | yes | biopsy | n/a | 9 |  |
| 19 | 49 | F | 1999 | IV | right colon | no | yes^c^ | resection | 5 | 23 |  |
| 20 | 64 | F | 2000 | IV | right colon | no | yes | biopsy | n/a | 16 |  |
| 21 | 65 | M | 2000 | IV | rectum | no | yes | biopsy | n/a | 33 |  |
| 22 | 56 | F | 1999 | IV | left colon | 5-FU, CPT-11 | yes | biopsy | n/a | 21 |  |
| 23 | 58 | M | 2001 | IV | right colon | no | yes | biopsy | n/a | 16 |  |
| 24 | 77 | F | 2004 | IV | rectum | no | yes^c^ | resection | 26 | 43 |  |
| 25 | 82 | M | 2009 | IV | right colon | 5-FU, Ox | no (M, 6 mos) | resection | 7 | 26 |  |
| 26 | 50 | M | 1989 | II | rectum | 5-FU | no (20 mos) | resection | 3 | 69 |  |
| 27 | 55 | F | 2007 | III | left colon | no | no (15 mos) | resection | 14 | 36 |  |
| 28 | 65 | F | 2004 | IV | rectum | no | yes^c^ | resection | 3 | 25 |  |
| 29 | 42 | F | 2006 | II | rectum | no | no (13 mos) | resection | >55 | >55 |  |
| 30 | 60 | F | 2008 | IV | rectum | 5-FU, Ox | no (M, 5 mos) | resection | 10 | >40 |  |
| 31 | 66 | M | 2007 | IV | rectum | 5-FU, Ox | no (5 mos) | resection | 7 | 14 |  |
| 32 | 53 | M | 2003 | II | rectum | no | no (27 mos) | resection | >56 | >96 |  |
| 33 | 42 | F | 1995 | IV | rectum | no | yes | biopsy | n/a | 2 |  |
| 34 | 59 | M | 2001 | IV | rectum | 5-FU | yes | biopsy | n/a | 13 |  |
| 35 | 60 | M | 1994 | IV | left colon | no | no (33 mos) | biopsy | n/a | 49 |  |
| 36 | 47 | M | 1996 | IV | rectum | no | no (7 mos) | resection | 26 | 74 |  |
| 37 | 43 | M | 2004 | IV | rectum | 5-FU, Ox^b^ | yes^c^ | resection | 3 | 72 |  |
| 38 | 64 | F | 1999 | IV | right colon | no | yes | biopsy | n/a | 10 |  |
| 39 | 46 | M | 2009 | IV | rectum | 5-FU, Ox | yes^c^ | resection | 3 | >32 |  |
| 40 | 65 | F | 1995 | IV | rectum | no | yes | biopsy | n/a | 28 |  |
| 41 | 73 | F | 2001 | IV | rectum | no | yes | biopsy | n/a | 20 |  |
| 42 | 75 | M | 2003 | II | rectum | no | no (13 mos) | resection | 9 | 53 |  |
| 43 | 76 | M | 1998 | III | right colon | no | no (12 mos) | resection | 13 | 54 |  |
| 44 | 40 | F | 1994 | IV | left colon | no | yes^c^ | biopsy | n/a | 58 |  |
| 45 | 68 | F | 2008 | IV | left colon | no | yes^c^ | resection | >30 | >30 |  |
| 46 | 60 | M | 2008 | IV | rectum | 5-FU, Ox, Beva | yes^c^ | resection | 8 | >24 |  |
| 47 | 45 | M | 2009 | IV | left colon | 5-FU, CPT-11 | yes^c^ | resection | 4 | >27 |  |
| 48 | 61 | F | 2000 | IV | right colon | no | yes | biopsy | n/a | 6 |  |
| 49 | 54 | F | 1998 | IV | left colon | no | yes | biopsy | n/a | 28 |  |
| 50 | 59 | M | 2001 | IV | left colon | no | no (108 mos) | resection | 25 | >124 |  |
| 51 | 46 | F | 2002 | IV | right colon | 5-FU^b^ | no (18 mos) | resection | 16 | 59 |  |
| 52 | 46 | F | 2002 | IV | rectum | no | yes^c^ | resection | >54 | >107 |  |
| 53 | 43 | F | 2002 | III | rectum | 5-FU^b^ | no (5 mos) | resection | 7 | 21 |  |
| 54 | 52 | F | 2002 | IV | left colon | 5-FU, CPT-11 | yes | resection | >72 | >110 |  |
| 55 | 50 | M | 2003 | III | right colon | 5-FU^b^ | no (31 mos) | resection | >59 | >107 |  |
| 56 | 72 | M | 2004 | III | rectum | no | no (12 mos) | resection | ? | 49 |  |
| 57 | 57 | M | 1998 | IV | right colon | no | no (28 mos) | resection | 30 | 58 |  |
| 58 | 34 | M | 2005 | IV | rectum | 5-FU, Ox^b^ | no (4 mos) | resection | 8 | >74 |  |
| 59 | 35 | F | 2006 | IV | left colon | 5-FU, Ox, Beva | yes | resection | 39 | 47 |  |
| 60 | 53 | M | 1995 | IV | rectum | 5-FU | yes | biopsy | n/a | 19 |  |
| 61 | 40 | M | 1999 | IV | left colon | no | yes | biopsy | n/a | 18 |  |
| 62 | 73 | F | 2008 | IV | left colon | no | no (4 mos) | resection | >27 | >40 |  |
| 63 | 60 | M | 1999 | IV | left colon | no | yes | resection | 3 | 33 |  |

^a^Right colon= cecum, ascending colon, hepatic flexure, transverse colon, splenic flexure; left colon= descending colon, sigmoid colon

^b^Chemotherapy before liver resection only. Surgeries on different dates within 3 months apart were considered synchronous. ^c^True synchronous (single operation). In cases classified as metachronous, removal of the primary preceded the metastasis except where indicated (M). The median interval between surgeries for metachronous pairs was 13 months. Abbreviations: dx, diagnosis; 5-FU, 5-flourouracil; CPT-11, irinotecan, Ox, oxaliplatin; beva, bevacizumab; n/a, not applicable; mos, months.

| Table S1B: Histopathologic Characteristics Including Percent of Cells Staining Positive for PTEN | | | | | |
| --- | --- | --- | --- | --- | --- |
| Patient | Lymph nodes | Histologic | Mucinous | Colon primary | Liver metastasis |
| No. | positive/total | differentiation (grade) | Features^a^ | % positive cells | % positive cells |
| 1 | 5/10 | intermediate/high | no | <10 | 0 |
| 2 | 8/12 | low | yes | 0 | 0 |
| 3 | 10/31 | low | no | 0 | 0 |
| 4 | 7/11 | high | yes | <5 | 0 |
| 5 | 5/9 | high | no | 0 | <10 |
| 6 | 0/20 | intermediate | no | 0 | 0 |
| 7 | 0/0 | intermediate | no | 0 | 75 |
| 8 | 1/12 | high | yes | 90 | 100 |
| 9 | 4/14 | intermediate/high | no | 100 | uninterpretable |
| 10 | 13/15 | intermediate | yes | 100 | 100 |
| 11 | NA | intermediate | no | 100 | 50 |
| 12 | 0/6 | intermediate/high | no | 100 | 100 |
| 13 | 3/11 | intermediate/high | no | 100 | 100; 70 |
| 14 | 14/15 | intermediate/high | no | >95 | 95 |
| 15 | 0/2 | intermediate/high | no | equivocal | 100 |
| 16 | 3/7 | intermediate | no | equivocal | 100 |
| 17 | 2/13 | intermediate | no | 100 | uninterpretable |
| 18 | 5/11 | intermediate | yes | 100 | 100 |
| 19 | 0/21 | intermediate/high | no | equivocal | 40 |
| 20 | 11/15 | intermediate | no | equivocal | 30 |
| 21 | 4/15 | intermediate | yes | 100 | 100 |
| 22 | 1/11 | intermediate | no | 100 | 100 |
| 23 | 4/4 | intermediate | no | 100 | 100 |
| 24 | 7/13 | high | no | 100 | 100 |
| 25 | 0/18 | intermediate | yes | 100 | 70 |
| 26 | 0/0 | intermediate | no | 100 | 95 |
| 27 | 5/8 | intermediate/high | no | 100 | 70 |
| 28 | 2/22 | intermediate/high | no | 100 | 100 |
| 29 | 0/8 | intermediate | no | 100 | 100 |
| 30 | 1/13 | low | yes | 100 | 100 |
| 31 | 8/14 | intermediate | no | 50 | 40 |
| 32 | 0/13 | low | no | 100 | 90 |
| 33 | 0/7 | high | no | 100 | 90 |
| 34 | 0/6 | high | yes | 100 | 100 |
| 35 | NA | intermediate | no | 100 | 100 |
| 36 | 0/1 | low | no | 100 | 100 |
| 37 | 0/1 | intermediate/high | no | 100 | 100 |
| 38 | 8/15 | intermediate | no | 100 | uninterpretable |
| 39 | 0/9 | intermediate | no | 100 | equivocal |
| 40 | 7/8 | intermediate | no | 100 | 70 |
| 41 | 1/5 | intermediate | no | 100 | 100 |
| 42 | 0/6 | intermediate/high | yes | 100 | 100 |
| 43 | 1/4 | intermediate | no | 100 | 80 |
| 44 | 3/7 | intermediate | yes | 100 | 100 |
| 45 | 1/19 | intermediate | no | 100 | 100 |
| 46 | 5/16 | intermediate | no | 100 | 100; 70 |
| 47 | 2/15 | low | yes | 100 | 100 |
| 48 | 1/4 | intermediate | no | 100 | 100 |
| 49 | 7/10 | intermediate | no | 100 | 100 |
| 50 | 0/12 | low/intermediate | no | 100 | 100 |
| 51 | 4/21 | intermediate | no | 100 | 100 |
| 52 | 1/7 | intermediate | no | 100 | 100 |
| 53 | 12/13 | high | no | 100 | 100 |
| 54 | 0/5 | intermediate | no | 100 | 100 |
| 55 | 3/52 | intermediate | no | 100 | 70 |
| 56 | 2/21 | low | no | 100 | 100 |
| 57 | 0/21 | intermediate | yes | equivocal | 70 |
| 58 | 20/20 | intermediate | yes | 100 | 100 |
| 59 | 14/31 | low | no | 100 | 90 |
| 60 | 4/34 | intermediate | no | 95 | uninterpretable |
| 61 | 0/11 | intermediate | no | 100 | uninterpretable |
| 62 | 6/22 | low | no | 100 | equivocal |
| 63 | 0/12 | intermediate | no | uninterpretable | 20 |

^a^Mucinous features identified in the primary tumor. Patient 18 was diagnosed with a mucinous adenocarcinoma (>50% of tumor cells exhibit mucinous features).

Table S2: Association of PTEN status with mutations in PIK3CA, RAS, and BRAF, among CRC primaries.^a^

|  |  |  | CRC primary | | | | |
| --- | --- | --- | --- | --- | --- | --- | --- |
|  |  |  | PIK3CA | |  | RAS/BRAF | |
|  |  |  | Wt | Mut |  | Wt | Mut |
| CRC primary | PTEN Neg |  | 7 (13.7) | 0 (0.0) |  | 3 (11.1) | 4 (13.8) |
|  | PTEN Pos |  | 44 (86.3) | 5 (100.0) |  | 24 (88.9) | 25 (86.2) |
| *P*-value for test of association | | | 1.00 | |  | 1.00 | |

^a^Stated figures are *N* (column percent); *p*-values are based upon Fisher’s exact test;

^a^Abbreviations: Neg, negative; Pos, positive; Wt, wild-type; Mut, mutant.

Table S3: Association of PTEN status with mutations in PIK3CA, RAS, and BRAF, among liver metastases.^a^

|  |  |  | Liver metastases | | | | |
| --- | --- | --- | --- | --- | --- | --- | --- |
|  |  |  | PIK3CA | |  | RAS/BRAF | |
|  |  |  | Wt | Mut |  | Wt | Mut |
| Liver metastases | PTEN Neg |  | 6 (12.2) | 0 (0.0) |  | 2 (7.7) | 4 (13.8) |
|  | PTEN Pos |  | 43 (87.8) | 6 (100.0) |  | 24 (92.3) | 25 (86.2) |
| *P*-value for test of association | | | 1.00 | |  | 0.672 | |

^a^Stated figures are *N* (column percent); *p*-values are based upon Fisher’s exact test;

^a^Abbreviations: Neg, negative; Pos, positive; Wt, wild-type; Mut, mutant.

Table S4: Summary of the fitted univariate Cox PH models.^a^

| Covariate | *N* | HR | 95% CI for HR | χ^2^ (df) | *p*-value |
| --- | --- | --- | --- | --- | --- |
| PTEN  Positive  Negative | 43  7 | 1.00  6.25 | (ref)  (1.98, 15.42) | 9.84 (1) | 0.0017 |
| PIK3CA  Wild-type  Mutant | 54  7 | 1.00  1.30 | (ref)  (0.49, 2.84) | 0.33 (1) | 0.564 |
| BRAS/RAF  Wild-type  Mutant | 29  34 | 1.00  2.03 | (ref)  (1.13, 3.71) | 5.79 (1) | 0.016 |
| Number of aberrations^b^ | 48 | 1.86 | (1.17, 2.89) | 6.96 (1) | 0.0083 |
| Aberration in PIK3CA or RAS/BRAF  No  Yes | 28  35 | 1.00  2.03 | (ref)  (1.13, 3.75) | 5.70 (1) | 0.017 |
| Age | 63 | 1.03 | (1.00, 1.05) | 4.59 (1) | 0.032 |
| Gender  Female  Male | 30  33 | 1.00  0.74 | (ref)  (0.42, 1.31) | 1.08 (1) | 0.300 |
| Stage at diagnosis  II  III  IV | 5  5  53 | 1.00  1.76  2.71 | (ref)  (0.38, 8.84)  (0.96, 11.11) | 4.02 (2) | 0.134 |
| Surgical timing  Metachronous  Synchronous^c^ | 22  41 | 1.00  2.86 | (ref)  (1.53, 5.53) | 11.54 (1) | 0.00068 |
| Chemotherapy before surgery  No  Yes, before liver surgery only  Yes, before both surgeries | 41  7  15 | 1.00  0.54  1.00 | (ref)  (0.19, 1.29)  (0.47, 1.97) | 1.91 (2) | 0.385 |
| Liver metastases resectable  Yes  No | 35  28 | 1.00  7.68 | (ref)  (3.81, 14.61) | 37.54 (1) | 9.0 x ${10}^{-10}$ |

^a^Abbreviations: χ^2^ = chi-square test statistic; df = degrees of freedom; ref = reference category.

^b^Amongst PTEN, PIK3CA, and BRAF/RAS; considered a linear predictor.

^c^ Colon and liver tumor samples collected within 3 months of each other.

Table S5: Summary of the fitted multivariate Cox PH model.^a^

| Covariate | *N* | HR | 95% CI for HR | $\chi_{1}^{2}$ | *p*-value |
| --- | --- | --- | --- | --- | --- |
| PTEN  Positive  Negative | 43  7 | 1.00  6.31 | (ref)  (2.03, 17.93) | 9.28 | 0.0023 |
| Age | 50 | 1.05 | (1.02, 1.09) | 8.69 | 0.0032 |
| Aberration in PIK3CA or BRAS/RAF  No  Yes | 22  28 | 1.00  2.11 | (ref)  (1.06, 4.39) | 4.55 | 0.033 |
| Liver metastases resectable  Yes  No | 30  20 | 1.00  8.91 | (ref)  (3.98, 21.11) | 28.55 | 9.2 x ${10}^{-8}$ |

^a^*N* = 50; the AUC at 10-year censoring was 0.90, and the c-index was 0.83;

the *p*-value = 0.80, for the global test of the null hypothesis: proportional hazards across time;

abbreviations: $\chi_{1}^{2}$ = chi-square test statistic with one degree of freedom; ref = reference category;

AUC = area under the ROC curve; ROC = receiver operating characteristic.

**Supplementary Figure Legends**

**Figure S1**. Kaplan-Meier estimates of overall survival of *A,* all patients in the cohort (N=63) and *B*, all patients by stratified presence of resectable or unresectable liver metastases.

**Figure S2** *A-B,* PIK3CA codon 545 allele frequencies in the primary tumor and liver metastasis from patient 13. *A*, in the primary sample, the adenine (A) peak area was below the threshold for calling a PIK3CA E545K mutation. *B*, in the metastasis, 19% of DNA had a guanine (G) to (A) base change, corresponding to a PIK3CA E545K mutation. *C-D*, KRAS codon 13 and PIK3CA codon 542 allele frequencies in the primary tumor and liver metastasis from patient 14. The PIK3CA mutant allele frequency was again below the level of detection in the primary and 19% in the metastasis; by contrast, in both tissues the KRAS mutant allele frequency was close to the expected 35% for macrodissected specimens containing 70% tumor cells with a heterozygous mutation. *C*, in the primary sample, 38% of DNA had a cytosine (C) to thymine (T) base change corresponding to a KRAS G13D mutation; the adenine (A) peak area was below the threshold for calling a PIK3CA E542K mutation. *D*, in the metastasis, 31% of DNA contained a (C) to (T) base change corresponding to a KRAS G13D mutation and 19% of DNA had a guanine (G) to (A) base change, corresponding to a PIK3CA E542K mutation. The left-most dashed lines denote un-extended primer. Other peaks in the KRAS spectra, indicated with gray dashed lines, result from multiplexing and are not part of the designated assays.

**Figure S3** Kaplan-Meier estimates of overall survival related to molecular markers in subcohorts of patients with unresectable or resected liver metastases. *A*, PTEN expression in colorectal primaries from patients with unresectable liver metastases; *B*, *C* any aberration in the PI3K or MAPK pathway in patients with unresectable (*B*) and resected (*C*) liver metastases. There were only two patients with PTEN null tumors in the liver resection subcohort (not shown).
