# Supplementary material for: PTEN expression is consistent in colorectal cancer primaries and metastases and associates with patient survival
Source: Cancer Med. 2013 Jun 10;2(4):496–506. doi: 10.1002/cam4.97 (PMC3799284; doi:10.1002/cam4.97)
Supplement: Supplementary file 6 [file cam40002-0496-SD6.doc]

**Figure S1**. Kaplan-Meier estimates of overall survival of *A,* all patients in the cohort (N=63) and *B*, all patients by stratified presence of resectable or unresectable liver metastases.

**Figure S2** *A-B,* PIK3CA codon 545 allele frequencies in the primary tumor and liver metastasis from patient 13. *C-D*, KRAS codon 13 and PIK3CA codon 542 allele frequencies in the primary tumor and liver metastasis from patient 14.

**Figure S3** Kaplan-Meier estimates of overall survival related to molecular markers in subcohorts of patients with unresectable or resected liver metastases.
